# Objectively Quantified Physical Activity and Sedentary Behavior in Predicting Visceral Adiposity and Liver Fat

**DOI:** 10.1155/2016/2719014

**Published:** 2016-09-29

**Authors:** Shelley E. Keating, Helen M. Parker, Toby G. Pavey, Michael K. Baker, Ian D. Caterson, Jacob George, Nathan A. Johnson

**Affiliations:** ^1^Faculty of Health Sciences, University of Sydney, Lidcombe, NSW, Australia; ^2^School of Human Movement and Nutrition Sciences, The University of Queensland, Brisbane, QLD, Australia; ^3^School of Exercise Science, Australian Catholic University, Sydney, NSW, Australia; ^4^Boden Institute of Obesity, Nutrition, Exercise and Eating Disorders, University of Sydney, Sydney, NSW, Australia; ^5^Storr Liver Centre, Westmead Millennium Institute and Westmead Hospital, University of Sydney, Westmead, NSW, Australia

## Abstract

*Objective*. Epidemiologic studies suggest an inverse relationship between nonalcoholic fatty liver disease (NAFLD), visceral adipose tissue (VAT), and self-reported physical activity levels. However, subjective measurements can be inaccurate and prone to reporter bias. We investigated whether objectively quantified physical activity levels predicted liver fat and VAT in overweight/obese adults.* Methods*. Habitual physical activity was measured by triaxial accelerometry for four days (*n* = 82). Time spent in sedentary behavior (MET < 1.6) and light (MET 1.6 < 3), moderate (MET 3 < 6), and vigorous (MET 6 < 9) physical activity was quantified. Magnetic resonance imaging and spectroscopy were used to quantify visceral and liver fat. Bivariate correlations and hierarchical multiple regression analyses were performed.* Results*. There were no associations between physical activity or sedentary behavior and liver lipid. Sedentary behavior and moderate and vigorous physical activity accounted for just 3% of variance for VAT (*p* = 0.14) and 0.003% for liver fat (*p* = 0.96). Higher levels of VAT were associated with time spent in moderate activity (*r* = 0.294, *p* = 0.007), but there was no association with sedentary behavior. Known risk factors for obesity-related NAFLD accounted for 62% and 40% of variance in VAT and liver fat, respectively (*p* < 0.01).* Conclusion*. Objectively measured levels of habitual physical activity and sedentary behavior did not influence VAT or liver fat.

## 1. Introduction 

The benefits of regular structured exercise are well documented and include improvements in cardiovascular and metabolic function, which reduce the risk of chronic health conditions such as type 2 diabetes and cardiovascular disease [1]. Recent evidence also demonstrates that exercise alone (in the absence of concomitant dietary restriction) reduces visceral adipose tissue (VAT) [2] and intrahepatic lipid (IHL) [3–5], both of which are independently associated with cardiovascular and metabolic risk [6]. While there is strong evidence for the benefits of regular structured exercise (i.e., exercise which is planned and involves repetitive movement), it is currently unclear whether increased levels of habitual activity (activity that is unstructured and accumulated during daily activities outside of structured exercise) and reduced sedentary behavior (activity with low metabolic rate (<1.6 METS) and in a reclining or seated position) have similar health benefits in the reduction of these adipose tissue depots. Understanding such associations has implications for policy as, because of low participation rates in structured exercise [7], there is an increasing emphasis in public health messages on the importance of habitual physical activity levels and reduced sedentary time [8].

Cross-sectional analyses indicate an inverse relationship between central adiposity as inferred by waist circumference [9], dual energy X-ray absorptiometry [10], or computed tomography (CT) [11] and self-reported physical activity levels. A similar inverse relationship has been observed for nonalcoholic fatty liver disease (NAFLD) [12–14]. Prospective studies have further supported this association [15–17]. In the Finnish Twin Cohort (TWINACTIVE) study, physically inactive cotwins were found to have liver fat scores (assessed via difference in mean signal intensity from magnetic resonance imaging) 170% greater than that of their active cotwin [17], and habitual physical activity, performed outside of work hours, was the strongest correlate of central abdominal fat after controlling for both environmental and genetic factors [18]. However, self-reported behavior is subject to reporter bias and tends to overestimate activity levels [19], especially in overweight and obese populations [20]. Emerging evidence using objectively measured physical activity via accelerometry supports these observations [21–24]; however research using accurate assessments of VAT and IHL is limited.

If the above epidemiological data [11–14] can be confirmed by high quality objective assessments, encouraging habitual physical activity and reducing sedentary behavior will have important clinical ramifications. Hence, in this study, we aimed to evaluate whether levels of accurately quantified sedentary behavior and habitual physical activity could predict levels of VAT and IHL in adults with overweight/obesity. Based on current guidelines for the management of obesity which recommend high volumes of moderate intensity activity [25], we hypothesised that there would be a negative relationship between time spent in moderate intensity physical activity and levels of VAT and IHL and a positive relationship between time spent in sedentary behavior and VAT and IHL content. Furthermore, we hypothesised that levels of sedentary behavior and moderate physical activity would explain a significant proportion of variance in VAT and IHL when modelled with known obesity-related health risk factors (including age, sex, body mass index (BMI), waist circumference, fasting glucose, fasting insulin, high density lipoprotein cholesterol, triglycerides, and resting blood pressure).

## 2. Methods

### 2.1. Participants

Participants were recruited via noticeboards, electronic bulletins, and clinical databases between June 2011 and January 2015 from a community-based sample of adult men and women (aged 18–60 years) with overweight or obesity (BMI ≥ 25.0 kg·m^−2^). Volunteers were excluded if they reported being physically active (exercising >3 days per week or meeting current guidelines for physical activity [1]) or were cigarette smokers. Participants provided clearance for participation from a medical practitioner prior to involvement and were excluded if there was evidence of an unstable cardiac condition, type 2 diabetes, hypertension, or liver disease other than nonalcoholic fatty liver disease (NAFLD) or secondary causes of steatohepatitis. Other exclusion criteria included taking lipid-lowering or insulin sensitizing medication, reporting taking *n*-3 polyunsaturated fatty acids within the last 6 months, or a change in medication within the last 12 months, regular alcohol consumption >20 g/day, or a significant change in body weight (>5% weight change) in the previous three months. The analysis included 82 volunteers from larger intervention trials (ANZCTRN12610000351011; ACTRN12614000723684), the results of which have been published elsewhere [5]. The study conformed to the ethical guidelines of the 1975 Declaration of Helsinki and was approved by The University of Sydney Human Research Ethics Committee and the Sydney Local Health District Ethics Review Committee. Eligible participants were screened via telephone interview and those meeting criteria provided informed consent and were invited to attend baseline assessments which included measures of anthropometry, a fasting blood test, magnetic resonance imaging (MRI) and spectroscopy (^1^H-MRS), and accelerometry.

### 2.2. Measurements

#### 2.2.1. Anthropometry and Blood Pressure Assessment

Stature was recorded by stadiometer (SECA model 220 Telescopic Height Rod, Hamburg, Germany). Weight was measured (Tanita BC-418 Body Composition Analyzer; Tanita Corporation, Tokyo, Japan) to the nearest 0.1 kg and BMI (kg·m^−2^) was calculated. Waist circumference was measured in triplicate at the horizontal plane, midway between the inferior margin of the ribs and the superior boarder of the iliac crest at end-expiration. Systolic (SBP) and diastolic (DBP) blood pressure were measured manually on each arm, after 10–15 min of quiet sitting.

#### 2.2.2. Biochemical Parameters

Venous blood was collected from the antecubital vein after an overnight fast (>10 hours). On the same day serum glucose, insulin, total cholesterol (TC), triglycerides (TG), high density lipoprotein cholesterol (HDL), low density lipoprotein cholesterol (LDL), alanine aminotransferase (ALT), aspartate aminotransferase (AST), and high sensitivity C-reactive protein (hs-CRP) were analysed by a commercially accredited laboratory.

#### 2.2.3. Magnetic Resonance Imaging (MRI) and Proton Magnetic Resonance Spectroscopy (^1^H-MRS)

Visceral adipose tissue was assessed via MRI and intrahepatic lipid via ^1^H-MRS using a 1.5 Tesla Achieva whole-body system (Philips Medical Systems; Best, Netherlands) as previously outlined [5, 26]. The cross-sectional areas for abdominal VAT were analysed using automated software (Hippo Fat*™*) [27] with manual editing as necessary. Total VAT volumes were calculated by summation of slice areas with adjustment for slice thickness. Liver spectra data were analysed by magnetic resonance user interface software (jMRUI version 4.0, EU Project) [26]. Hepatic water signal amplitude was measured from the nonwater suppressed spectrum [5].

#### 2.2.4. Objective Activity Monitoring

Current mean time spent in physical activity, sedentary behavior, daily steps, and energy expenditure were assessed by triaxial accelerometer (SenseWear*™*, BodyMedia Inc., PA, USA). Data were collected from all participants for four days (3 weekdays and 1 weekend, which was the minimum inclusion criteria) for 24 hours per day, except during water-based activities including showering, bathing, or swimming. All data were collected at least 7 days after magnetic resonance imaging. Armbands were worn on the left upper arm. Participants were instructed to continue with their habitual routine during this period. Physical activity levels and sedentary behavior were quantified (BodyMedia software, v8.0) with sedentary behavior defined as <1.6 METs, “light” activity as 1.6 < 3 METs, “moderate” activity as 3 < 6 METs, “vigorous” activity as 6 < 9 METs, and “high” activity as ≥9 METs [28]. Sleep duration was not included in time spent in sedentary behavior. Data were included if the monitor was worn for >85% of a 24-hour day.

### 2.3. Statistical Analysis

All descriptive data are reported as means ± standard error from the mean (SE). Differences between groups were compared by Student's *t*-test with homogeneity of variances assessed via Levene's test for equality of variance or *χ* ^2^ for categorical data. Pearson coefficients (*R*) were used for all correlations. Separate hierarchical multiple regression analyses were performed for prediction of VAT and IHL percentage. Simple anthropometric and demographic variables were entered at Block 1 for both VAT and IHL. Known cardiometabolic risk variables were entered into the regression at Block 2, based on the variables that were significantly associated with VAT and IHL, respectively, in our sample (Table 2). These included TG, glucose, insulin, SBP, DBP, ALT and BMI for VAT and glucose, insulin, ALT, HDL, and BMI for IHL. While TG was not significantly associated with VAT in our analysis, it was included given its previously established association with excess VAT [29]. Finally, mean time spent in sedentary behavior, moderate intensity activity, and vigorous intensity activity were entered into Block 3 of the regression analysis. Data were analysed using Statistical Package for the Social Sciences (SPSS version 22.0; IMB Corp., Armonk, NY, USA); *p* values were based on two-sided tests and considered statistically significant at *p* < 0.05.

## 3. Results

### 3.1. Participants

Eighty-two adults (60% male) participated in the study. Mean age was 40.1 ± 1.9 years, BMI 30.9 ± 0.5 kg·m^−2^, waist circumference 100.0 ± 1.1 cm, and liver fat 5.3 ± 0.5%. Thirty-five percent of the participants were classified as having NAFLD based on IHL > 5.5% [30]. Other participants' characteristics are summarised in Table 1. Mean device wear time was 97 ± 2% of a 24-hour day. Participants with NAFLD had a significantly higher BMI, waist circumference, SBP, VAT, fasting glucose, and fasting insulin (*p* < 0.05). There were no differences in objectively measured levels of physical activity, sedentary behavior, total daily energy expenditure, or the number of steps taken per day between participants with and without NAFLD (Table 1). Time spent in high intensity activity (>9 METS) was negligible (mean 37 ± 27 seconds) with only 3/82 participants (4%) engaging in high intensity activity for more than an average of one minute in total. When high intensity activity was included in the analysis, the results were not altered (data not shown). While there are no established critical thresholds for VAT volume with respect to morbidity and mortality risk, based on gender specific waist circumferences for Caucasian populations, 51% of men and 73% of women were above the risk threshold for abdominal obesity [31].

### 3.2. Bivariate Correlations

Bivariate correlations between VAT and IHL and biochemical, anthropometric, and accelerometry assessed activity variables are summarised in Table 2. A higher volume of VAT was associated with higher IHL, BMI, waist circumference, SBP, DBP, fasting glucose, fasting insulin, and ALT (Table 2). There was a weak association between higher levels of VAT and higher amounts of moderate intensity physical activity (*r* = 0.294, *p* = 0.007). Higher levels of IHL were associated with higher BMI, waist circumference, VAT, fasting glucose, and fasting insulin and lower levels of HDL, but there was no association between IHL and total energy expended, time spent in sedentary behavior, and time spent in any level of activity (Table 2).

### 3.3. Hierarchical Multiple Regression Analysis

Demographic and anthropometric variables (waist circumference, sex, and age) accounted for 54% and 30% of the variance in VAT and IHL, respectively (*p* < 0.001; Tables 3 and 4). The addition of TG, glucose, insulin, SBP, DBP, and BMI in Block 2 accounted for a further 9% of variance in VAT (*p* = 0.032). The addition of glucose, HDL, ALT, insulin, and BMI increased the prediction of IHL to 40%; however this was not significant (*p* = 0.06). The addition of time spent in sedentary behavior, moderate and vigorous intensity activity (Block 3), increased the prediction of VAT to 65% (*p* = 0.14) and did not change the prediction of IHL (*p* = 0.96). Waist circumference, triglyceride, and fasting glucose contributed significantly to the prediction of high levels of VAT while waist circumference and fasting insulin contributed significantly to the prediction of high levels of IHL.

## 4. Discussion

We aimed to evaluate whether levels of accurately quantified sedentary behavior and habitual physical activity could predict levels of visceral adiposity and liver fat. Surprisingly, while traditional risk factors for cardiovascular disease (e.g., sex, age, and waist circumference) were associated with VAT and IHL, no relationship was observed between objectively measured physical inactivity and raised liver fat. Further, we unexpectedly observed a weak positive association between VAT volume and time spent in moderate activity (i.e., increased VAT was associated with higher time spent in moderate intensity physical activity) and also total energy expenditure. The inclusion of objectively measured levels of moderate activity and sedentary behavior did not improve the potency of traditional risk factors (waist circumference, triglycerides, fasting glucose, or fasting insulin) for predicting the liver fat concentration or level of visceral adiposity. As a cross-sectional analysis, this study demonstrates that current habitual activity levels are not related to VAT or liver fat; however it does not account for lifetime levels of habitual activity. Further, while objective assessment of activity via accelerometry is considered more accurate than self-report methods, participants may still have altered their usual behavior during monitoring. While a significant reduction in liver fat has been observed with just 4 weeks of structured aerobic exercise [32], it is uncertain how long current habitual activity patterns may take to affect VAT and liver fat.

On the basis of epidemiological data at the population level, physical inactivity is associated with the development of NAFLD [12–14] and visceral adiposity [11], and reducing sedentary behavior (including sitting time) should be generally advocated in all populations and is now recognized in public health messages [8]. However, our findings suggest that objectively measured physical activity is not greatly predictive of risk at an individual level and the associations between habitual physical activity levels, sedentary behavior, and metabolically detrimental fat depots such as VAT and IHL remain uncertain. While our findings appear counterintuitive to epidemiological data, unlike these studies, we accurately quantified both liver fat and VAT, as well as 24-hour habitual physical activity behavior, suggesting that the relationship may not be as straightforward.

There is now strong and consistent evidence that regular structured physical activity (exercise training) reduces VAT [2] and IHL [3–5]. This reflects current physical activity guidelines which advocate structured exercise for health benefits [1]. With the less potent stimulus of general habitual activity, the volume of activity required for benefit is much greater (e.g., the accumulation of >470 min of physical activity for weight loss [25]). While increasing incidental activity is important for increasing total daily energy expenditure, there is an established dose potency of structured exercise for a range of cardiovascular and metabolic health risk variables [1]. Additionally, there are well-established inverse relationships between cardiorespiratory fitness and NAFLD [33], histological severity of liver disease [34], and VAT [35]. Indeed high levels of cardiorespiratory fitness is a characteristic of the phenomenon termed “metabolically healthy obesity” [36] and low cardiorespiratory fitness is an independent predictor of all-cause and cardiovascular disease mortality [37]. In consideration of this, the emphasis for behavior change in this population should arguably be on adopting and adhering to structured exercise programs, cognizant of individual barriers to participation in exercise and exercise preferences, which may require facilitation with behavioral management strategies.

Obesity in developed nations is linked with an obesogenic environment which encompasses a calorie-dense diet (overnutrition) and a physical activity hostile (or physical inactivity-promoting) environment. However, while both physical inactivity and excess adiposity are independently associated with a greater risk of mortality [38], it is contentious whether physical inactivity is a cause or an effect of obesity [39, 40]. Therefore, while it is established that low amounts of physical activity and high amounts of sedentary behavior predict mortality [41], the role of these behaviors in the development of abdominal obesity and liver fat is unclear and perhaps minimal.

Recent research has suggested that prolonged sitting (≥8 hours/day) is associated with an increased risk of all-cause mortality [41, 42] and type 2 diabetes [42]. However, in support of our findings, the association between sedentary behavior and markers of cardiometabolic risk disappear when adjusted [43] or modified [42] for total physical activity levels. Hence only individuals with low activity and high levels of sedentary behavior such as sitting may be at increased health risk [44]. While there is a heightened understanding of the detrimental effects of increased sedentary behavior and sitting time [42, 45] and interrupting sitting time may confer some health benefit, this study suggests that a direct link between unstructured, objectively measured levels of physical activity and sedentariness and levels of metabolically risky fat depots is insignificant.

In concordance with these findings, despite significant associations with moderate-to-vigorous physical activity, no associations were observed between objectively quantified sedentary time and hepatic steatosis (estimated via CT) in adults with NAFLD [23] or with VAT volume in men or women [46]. A large analysis in 3056 volunteers observed that individuals with NAFLD (defined by the fatty liver index [47]) spent less time in light, moderate, or vigorous activity than non-NAFLD counterparts and that the lowest quartile of physical activity was found in individuals with NAFLD and comorbid type 2 diabetes [22]. It has also been observed that individuals meeting 150 minutes/week of moderate-to-vigorous physical activity as assessed via accelerometry had the lowest odds of hepatic steatosis (assessed via CT) independent of BMI (OR 0.77) [23]. However, in these investigations, it is unclear whether the low levels of physical activity caused the elevated liver fat content or whether NAFLD itself caused patients to have low participation in physical activity.

A limitation of this cross-sectional analysis is that the non-NAFLD cohort had low mean liver fat levels which reduced the range in the data. An important consideration is that we recruited adults with overweight/obesity who had no known comorbid cardiovascular or metabolic disease, which probably contributed to this limited spread. Further research examining the interaction between sedentary behavior, physical activity levels, and metabolically detrimental fat depots, employing gold-standard methods, across a more diverse population, is therefore warranted. Another limitation is that the aforementioned associations between physical activity, fitness, and NAFLD may not translate to disease severity. A retrospective analysis of 813 adults with biopsy-proven NAFLD demonstrating that only those who reported meeting the minimal guidelines for vigorous intensity activity (≥75 min/week) and not moderate physical activity guidelines (150 to ≥300 min/week) reduced the odds of developing NASH (OR 0.65) with further reduced odds (OR 0.56) when the extensive guidelines for vigorous intensity activity (≥150 min/week) were met. Furthermore, the odds of advanced fibrosis were only reduced in patients meeting the extensive guidelines for vigorous activity [48]. These data suggest that vigorous activity* per se* may be necessary for improvements in the progressive forms of NAFLD although longitudinal data from clinical trials are lacking and limited by the invasive nature of liver biopsy to inform disease severity.

In conclusion, there were no associations between sedentary behavior and levels of IHL or VAT and low levels of objectively measured physical activity did not predict high levels of VAT or IHL. These findings suggest that while traditional risk factors such as waist circumference, triglycerides, fasting glucose, or fasting insulin may predict the incidence of NAFLD or high levels of VAT, objectively quantified physical activity levels and sedentary behavior did not predict those at risk.

## Figures and Tables

**Table 1:** Participants' characteristics.

	Total group (*n* = 82)	NAFLD (*n* = 29)	Non-NAFLD (*n* = 53)	*p* ^*∗*^
*Demographics and anthropometry *				
Age (years)	40.1 (1.9)	41.7 (2.3)	39.3 (1.4)	0.34
Sex (M/F)	49/33	18/11	31/22	0.75
BMI (kg·m^−2^)	30.9 (0.5)	32.7 (1.1)	29.9 (0.5)	0.02
Waist circumference (cm)	100.0 (1.1)	105.8 (2.0)	96.8 (1.2)	<0.001
SBP (mmHg)	127.4 (1.5)	132.1 (2.7)	124.8 (1.8)	0.02
DBP (mmHg)	81.0 (0.8)	83.1 (1.3)	79.8 (1.0)	0.05
SAT (cm^3^)	9268 (405)	10431 (806)	8632 (427)	0.55
VAT (cm^3^)	3153 (169)	3847 (301)	2774 (185)	0.004
IHL (%)	5.3 (0.5)	10.3 (0.9)	2.6 (0.2)	<0.001
*Biochemistry *				
Glucose (mmol/L)	4.3 (0.1)	4.6 (0.1)	4.1 (0.1)	<0.001
Insulin (mU/L)	8.4 (0.5)	10.6 (1.2)	7.2 (0.5)	0.01
ALT (U/L)	27.9 (1.5)	31.8 (2.2)	25.8 (1.8)	0.05
AST (U/L)	24.7 (1.1)	25.9 (1.8)	24.0 (1.3)	0.40
hsCRP (mg/L)	8.4 (0.5)	4.6 (0.9)	2.7 (0.4)	0.73
*Lipids*				
Total cholesterol (mmol/L)	5.5 (0.1)	5.5 (0.2)	5.5 (0.1)	0.99
Triglyceride (mmol/L)	1.3 (0.1)	1.6 (0.2)	1.2 (0.1)	0.05
LDL (mmol/L)	3.5 (0.1)	3.5 (0.2)	3.5 (0.1)	0.99
HDL (mmol/L)	1.4 (0.0)	1.3 (0.1)	1.5 (0.0)	0.06
*Habitual physical activity*				
Sedentary behavior (<1.6 METs) (hours:min)	12:08 (0:12)	12:10 (0:20)	12:08 (0:16)	0.93
Light physical activity (1.6–2.9 METs) (hours:min)	2:58 (0:06)	2:48 (0:11)	3:04 (0:07)	0.20
Moderate physical activity (3–5.9 METs) (hours:min)	1:30 (0:07)	1:34 (0:15)	1:29 (0:06)	0.75
Vigorous physical activity (6–8.9 METs) (hours:min)	0:05 (0:01)	0:04 (0:01)	0:06 (0:0)	0.39
Steps (per day)	8915 (355)	8855 (751)	8947 (371)	0.90
Total energy expenditure (kJ)	13061 (485)	13145 (541)	13015 (693)	0.90

M: male; F: female; BMI: body mass index; NAFLD: nonalcoholic fatty liver disease; SBP: systolic blood pressure; DBP: diastolic blood pressure; SAT: subcutaneous adipose tissue; VAT: visceral adipose tissue; IHL: intrahepatic lipid; ALT: alanine aminotransferase; AST: aspartate aminotransferase; hsCRP: high-sensitivity c-reactive protein; LDL: low density lipoprotein cholesterol; HDL: high density lipoprotein cholesterol; and METs: metabolic equivalents. Values are mean (standard error). ^*∗*^ *p* value for NAFLD versus non-NAFLD using Student's *t*-test for continuous variables and *χ* ^2^ for categorical variables.

**Table 2:** Descriptive statistics and correlations of metabolic, anthropometric, and accelerometry measured physical activity outcomes.

	1	2	3	4	5	6	7	8	9	10	11	12	13	14	15	16	17	18
(1) IHL%																		
(2) BMI	0.363^*∗∗*^																	
(3) Waist	0.539^†^	0.612^†^																
(4) SBP	0.089	−0.004	0.337^*∗∗*^															
(5) DBP	0.066	0.018	0.288^*∗∗*^	0.625^†^														
(6) Glucose	0.270^*∗*^	0.207	0.340^*∗∗*^	0.237^*∗*^	0.195													
(7) Insulin	0.462^†^	0.498^†^	0.468^†^	0.009	0.021	0.298^*∗∗*^												
(8) ALT	0.297^*∗∗*^	−0.043	0.440^†^	0.395^†^	0.254^*∗*^	0.209	0.193											
(9) AST	0.147	−0.072	0.261^*∗*^	0.261^*∗*^	0.114	−0.043	−0.019	0.681^†^										
(10) TG	0.213	−0.084	0.079	0.309^*∗∗*^	0.293^*∗∗*^	0.238^*∗*^	0.165	0.110	0.025									
(11) HDL	−0.256^*∗*^	−0.009	−0.216	−0.017	0.004	−0.042	−0.248^*∗*^	−0.162	−0.038	−0.248^*∗*^								
(12) hsCRP	0.216	0.503^†^	0.244^*∗*^	−0.042	0.013	0.114	0.244^*∗*^	−0.098	−0.172	−0.065	0.054							
(13) VAT	0.385^†^	0.320^*∗∗*^	0.666^*∗∗*^	0.374^*∗∗*^	0.318^*∗∗*^	0.419^†^	0.247^*∗*^	0.371^*∗∗*^	0.209	0.384	−0.211	0.120						
(14) TEE	0.075	−0.034	0.193	0.193	0.133	−0.005	0.003	0.167	0.177	0.023^†^	−0.212	−0.163	0.342^*∗∗*^					
(15) Steps	−0.050	−0.108	0.182	0.182	0.125	−0.097	−0.124	0.173	0.285^*∗∗*^	0.076	−0.183	−0.099	0.170	0.298^*∗∗*^				
(16) Sed	0.026	0.410^†^	−0.007	−0.345^*∗∗*^	−0.138	0.107	0.109	−0.356^*∗*^	−0.286^*∗∗*^	−0.126	0.079	0.234^*∗*^	−0.117	−0.342^*∗∗*^	−0.457^†^			
(17) Light	−0.72	−0.379^†^	−0.060	0.103	0.023	−0.122	−0.268^*∗∗*^	0.156	−0.048	0.116	−0.080	−0.310^*∗∗*^	0.138	0.315^*∗∗*^	0.415^*∗∗*^	−0.793		
(18) Mod	−0.024	−0.363^*∗∗*^	0.124^†^	0.383^†^	0.215	−0.081	0.172	0.291^*∗∗*^	0.353^*∗∗*^	0.144	−0.119	−0.285^*∗∗*^	0.294^*∗∗*^	0.487^†^	0.692^†^	−0.879^†^	0.507^†^	
(19) Vig	−0.126	−0.352^*∗∗*^	−0.101^†^	0.295^*∗∗*^	0.174	0.15	−0.268^*∗*^	0.136	0.039	−0.130	0.191	−0.234^*∗*^	−0.049	0.114	0.123	−0.372^*∗∗*^	0.244^*∗*^	0.323^*∗∗*^

^*∗*^ *p* < 0.05;  ^*∗∗*^ *p* < 0.01; and  ^†^ *p* < 0.001.

Data presented are the correlation coefficient (*r*). IHL: intrahepatic lipid; BMI: body mass index; SBP: systolic blood pressure; DBP: diastolic blood pressure; ALT: alanine aminotransferase; AST: aspartate aminotransferase; TG: triglyceride; HDL: high density lipoprotein cholesterol; hsCRP: high-sensitivity c-reactive protein; VAT: visceral adipose tissue; TEE: total energy expenditure; Sed: sedentary behavior; Light: light intensity physical activity; Mod: moderate intensity physical activity; and Vig: vigorous intensity physical activity.

**Table 3:** Hierarchical regression model for prediction of visceral adiposity.

	*R*	*R* ^2^	Adjusted *R* ^2^	*R* ^2^ change	*p*	*β*
*Block 1*	0.732	0.536	0.518	0.536	<0.001	
Sex						−0.251
Age						**0.386**
Waist						**0.503**
*Block 2*	0.790	0.624	0.571	0.087	0.032	
Sex						−0.148
Age						**0.244**
Waist						**0.525**
TG						**0.264**
SBP						0.029
DBP						−0.36
ALT						0.052
BMI						0.001
Glucose						0.127
Insulin						−0.094
*Block 3*	0.808	0.653	0.587	0.029	0.136	
Sex						−0.022
Age						0.194
Waist						**0.510**
TG						**0.241**
SBP						−0.039
DBP						−0.005
ALT						0.062
BMI						0.056
Glucose						**0.174**
Insulin						−0.086
Sedentary						−0.031
Moderate						0.212
Vigorous						−0.023

BMI: body mass index; SBP: systolic blood pressure; DBP: diastolic blood pressure; ALT: alanine aminotransferase; and TG: triglyceride. Boldface indicates statistical significance (*p* < 0.05).

**Table 4:** Hierarchical regression model for prediction of liver fat.

	*R*	*R* ^2^	Adjusted *R* ^2^	*R* ^2^ change	*p*	*β*
*Block 1*	0.550	0.303	0.276	0.303	<0.001	
Sex						0.102
Age						0.026
Waist						**0.575**
*Block 2*	0.630	0.397	0.331	0.094	0.055	
Sex						0.176
Age						0.114
Waist						**0.471**
Glucose						0.23
HDL						−0.162
ALT						0.104
BMI						−0.146
Insulin						**0.256**
*Block 3*	0.632	0.400	0.305	0.003	0.957	
Sex						0.166
Age						0.136
Waist						**0.481**
Glucose						0.008
HDL						−0.180
ALT						0.105
BMI						−0.165
Insulin						**0.260**
Sedentary						0.059
Moderate						0.000
Vigorous						0.046

BMI: body mass index; ALT: alanine aminotransferase; and HDL: high density lipoprotein cholesterol. Boldface indicates statistical significance (*p* < 0.05).

## Data Access

Shelley E. Keating and Nathan A. Johnson have access to all of the data and can vouch for the integrity of the data analyses.
